# Balancing harm and harmony: Evolutionary dynamics between gut microbiota-derived flagellin and TLR5-mediated host immunity and metabolism

**DOI:** 10.1080/21505594.2025.2512035

**Published:** 2025-05-30

**Authors:** Boram Seo, Mi Young Lim

**Affiliations:** Precision Nutrition Research Group, Food Functionality Research Division, Korea Food Research Institute, Wanju-gun, Jeollabuk-do, Republic of Korea

**Keywords:** Flagellin, TLR5 signalling, gut microbiota, immune modulation, metabolic homoeostasis, host-microbe interaction

## Abstract

The gut microbiota maintains host health and shapes immune responses through intricate host-microbe interactions. Bacterial flagellin, a key microbe-associated molecular pattern, is recognized by Toll-like receptor 5 (TLR5) and NOD-like receptor family caspase activation and recruitment domain-containing 4 inflammasome. This dual recognition maintains the delicate balance between immune tolerance and activation, thereby influencing health and disease outcomes. Therefore, we explored the structural and functional evolution of bacterial flagellin to elucidate its role in innate and adaptive immune responses, along with its impact on metabolic processes, particularly via TLR5. In this review, we highlight the diagnostic and therapeutic potential of flagellin, including its application in vaccine development, cancer immunotherapy, and microbiome-based therapies. We integrated perspectives from structural biology, immunology, and microbiome research to elucidate the co-evolutionary dynamics between gut microbiota-derived flagellin and host immunity. Our interpretations provide a basis for the development of innovative strategies to improve health and disease management.

## GRAPHICAL ABSTRACT

**Figure:**
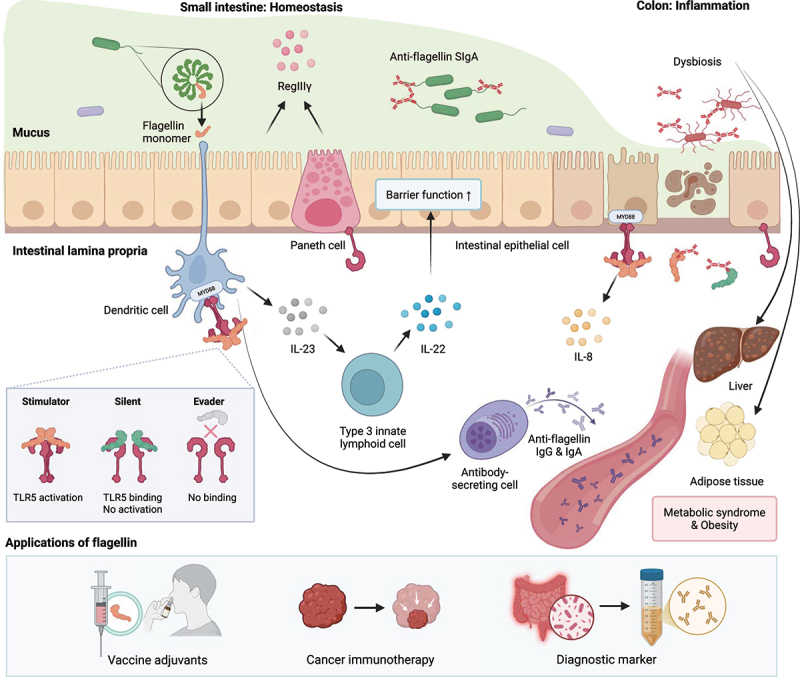

## Introduction

The gut microbiota is a highly dynamic and complex ecosystem comprising trillions of microorganisms, including bacteria, archaea, fungi, and viruses [1,2]. Bacteria account for most of the biomass in the gut flora; they maintain host health and immune regulation [3,4]. Gut bacteria enable the host immune system to distinguish pathogens from commensals through microbe-associated molecular patterns (MAMPs) or pathogen-associated molecular patterns (PAMPs) [5–7]. This prevents overreaction of the immune system, which could lead to unnecessary inflammation. Dysbiosis occurs when the gut microbiota is disrupted, potentially leading to various diseases such as inflammatory bowel disease (IBD) and metabolic disorders, including obesity, type 2 diabetes, metabolic dysfunction-associated steatotic liver disease, and malnutrition [8–10].

The interaction between gut bacteria and the host immune system has been shaped by co-evolution over time, resulting in a finely tuned balance between immune tolerance and defence [11–13]. The host immune system is central to this balance, and its response is broadly divided into innate and adaptive immunity. Innate immunity serves as the first line of defence, delivering an immediate yet non-specific response to pathogens. The key components of this immune response include physical barriers, phagocytic cells, and pattern recognition receptors (PRRs), such as Toll-like receptors (TLRs) and NOD-like receptors (NLRs) [14,15]. Although PRRs have conventionally been associated with the detection of PAMPs, their ability to bind ligands from microbiota has been increasingly recognized [7]. Adaptive immunity can recognize specific antigens to generate a precise response that is predominantly orchestrated by lymphocytes and immunoglobulins. It provides long-term immunity and immunological memory, allowing the host to respond more efficiently to subsequent exposures to the same antigen [16].

Advancements in gut microbiome research have facilitated the development of beneficial bacterial strains that are closely associated with human health into next-generation probiotics and personalized medicinal products [17,18]. However, the industrial production and commercialization of these obligate anaerobes face significant challenges, including difficulties in mass production and formulation of viable cells [19,20]. Gut microbiota-derived substances, such as metabolites and proteins, may mitigate these limitations [21–23]. For example, commensal bacteria-derived proteins such as flagellin demonstrate the therapeutic potential of gut microbiota-derived substances, as they modulate immune responses [24].

Bacterial flagellin, the protein that constitutes the filament of bacterial flagella, is a key factor in host-microbe interactions [24,25]. Flagellin is a MAMP produced by diverse bacterial phyla, including both gram-negative Proteobacteria (Pseudomonadota) and gram-positive Firmicutes (Bacillota) [26]. This protein not only plays a role in bacterial motility but also has a substantial impact on host immune responses. It is recognized by innate immune response mechanisms through TLR5 in the extracellular space and NLR family caspase activation and recruitment domain-containing protein 4 (NLRC4) inflammasome in the cytosol. This dual recognition triggers immune responses that can lead to both inflammation and immune tolerance [27–29]. TLR5 signalling activates the NF-κB pathway, consequently driving pro-inflammatory cytokine production, whereas NLRC4 induces interleukin-18 (IL-18) secretion via inflammasome activation [30]. Although these responses primarily clear infections, they can also contribute to chronic inflammation if poorly regulated [31].

TLR5 is primarily recognized for its ability to detect flagellin from pathogens such as *Salmonella*. However, the main source of flagellin in the human gut is the Lachnospiraceae family within Firmicutes [32]. Therefore, the recognition and regulation of flagellated gut microbiota significantly influence the modulation of host immunity.

Variations in flagellin structure and function reflect an evolutionary race, with bacteria evolving to evade immune detection or stimulate a tolerogenic response to persist within the host [33,34]. This dynamic interplay highlights the intricate co-evolution of bacterial virulence factors and host immune response mechanisms. In this review, we summarize the structural evolution of bacterial flagellin and its intricate interactions with both the innate and adaptive immune response mechanisms. We further demonstrate their roles in metabolic regulation, potential as diagnostic biomarkers, and applications in microbiome-based therapies. We evaluated the evolutionary dynamics between gut bacterial flagellin and host immunity to uncover the complex co-evolutionary processes that shape host-microbe interactions and their profound implications for health and disease.

## Structural and evolutionary dynamics of bacterial flagellin

### Structure and functions of flagellin

Bacterial flagellin is a primary protein that forms the structural backbone of flagella, thereby enabling motility and contributing to colonization across various environments [26]. The flagellin monomer is structured into four main domains: the D0 and D1 domains are helical, whereas the D2 and D3 domains are globular [27,35,36]. These domains are consistent across a diverse range of bacterial species, with the N- and C-terminal D0-D1 regions being particularly conserved [27,37]. These conserved regions maintain the structural stability of the flagellar filament and facilitate interaction with the host innate immune response systems, specifically through recognition by TLR5.

The flagellum comprises a flagellar filament connected to a molecular motor system that includes the transmembrane basal body and hook complex. It facilitates bacterial navigation in various host environments [38–40]. The inner D0 and D1 domains form an elongated helical rod structure that maintains the structural integrity of the flagellum [41]. The conserved N- and C-terminals within the D0/D1 domain contribute to the structural assembly of the flagellum and to the functional flexibility required for effective motility [42]. The N-terminal of the D1 domain contains the epitope recognized by TLR5, a MAMP that is critical for initiating the immune response [27].

The ability of TLR5 to recognize flagellin is highly specific to its monomeric form, as the TLR5 epitope is buried within the flagellar filament structure and is inaccessible in its polymerized state [36]. The D1 domain is essential for high-affinity binding and effective TLR5 signalling, whereas the D0 domain performs a complementary function in the activation of TLR5, albeit with minimal impact on binding [41,43]. This structural arrangement elucidates the coordinated roles of the D0 and D1 domains in both structural assembly and immune recognition. The immunostimulatory activity of flagellin was preserved even after extensive truncation of the hypervariable D2/D3 region using recombinant flagellins with targeted deletions [44]. These deletions markedly reduce antigenicity while maintaining TLR5 activation capacity. This demonstrates the dominant role of the conserved D0/D1 domains in innate immune stimulation and structural integrity [44].

Studies on *Salmonella typhimurium* further illustrate the close relationship between the flagellin structure and its role in motility [45,46]. For instance, alanine point mutations in key residues that affect the convex surface of the flagellin D1 domain disrupt both TLR5 recognition and motility [27]. Moreover, removal of up to 100 amino acids from the variable D2/D3 region does not abolish flagellar movement. This indicates that these domains are not essential for fundamental motility functions [46]. However, larger deletions lead to a near-complete loss of motility and structural stability, indicating that the D2/D3 domains provide additional support for flagellar function [46].

The outer D2 and D3 domains are mostly composed of β-strands and exhibit high variability among bacterial species. This variability is indicative of evolutionary adaptations that allow bacteria to avoid immune detection by altering parts of the flagellin protein exposed to the host [27,47]. The variability of the D3 domain, which is more surface-exposed, is particularly important for evading recognition by the host antibodies. The hypervariable region (HVR) of flagellin differs considerably even within the same species, depending on whether the strain is symbiotic. For instance, the symbiotic *Escherichia coli* Nissle 1917 strain has an extended HVR that strongly activates TLR5 compared to commensal strains. This difference in HVR length and composition underscores the effect of flagellin variability on bacterial interactions with the host immune system and their ecological roles [48].

Not all bacterial flagellins possess the D2 and D3 domains [49]. For instance, the flagellin of *Ligilactobacillus ruminis* lacks these variable outer domains and relies on conserved core regions to fulfill its structural and functional roles [50]. Similarly, approximately 50% of bacterial flagellins lack the D2/D3 domains. Instead, they rely entirely on the conserved D0/D1 regions for essential functions such as motility and immune recognition [51]. These findings highlight an evolutionary adaptation that allows motility, even in the absence of outer domains. This provides additional insights into the streamlined architecture of certain bacterial species while minimizing immune detection. This streamlined architecture illustrates alternative evolutionary strategies for maintaining essential functionality and evading host immune responses.

Recent morphological studies have further demonstrated the adaptability of flagellar filaments. The D2/D3 domain plays a role in dimerization, whereas the core D0 and D1 interactions remain unaffected [49]. These findings illustrate the polymorphic transformation of flagellar filaments. This process is crucial for their assembly into protofilaments, and into the dynamic adjustments required for bacterial motility. Amino acid replacement in flagellin and environmental changes can disrupt this assembly. These disruptions can substantially impair bacterial motility, consequently affecting the ability of bacteria to colonize and survival in the host [52–54].

The regulatory mechanisms underlying flagellin synthesis play a critical role in the modulation of motility during infection. For example, *Salmonella enterica* employs a trans-acting leader RNA derived from its virulence operon to suppress flagellin synthesis in the host macrophages [55]. This suppression limits bacterial motility and reduces TLR5-mediated immune detection while maintaining the flexibility to resume motility under favourable conditions. Such mechanisms illustrate the evolutionary balance between motility and immune evasion, highlighting the dual role of flagellin in bacterial survival and host interactions.

The structural and functional features of flagellin underscore its biological significance. As flagellin adapts to selective pressure to maintain motility while evading immune recognition, it has evolved diverse antigenic profiles that shape its interactions with TLR5. This intricate co-evolution of bacterial flagellin and host immune receptors has profound implications for immune detection and bacterial functionality.

### Evolutionary dynamics of flagellin

The evolutionary adaptation of flagellin is shaped by the preservation of motility and evasion of immune responses, which significantly influence its structural and functional roles across bacteria. This adaptation has led to diverse antigenic profiles in flagellin hypervariable regions, allowing species to avoid immune detection while maintaining crucial motility [26]. The interaction between flagellin and TLR5 enables the immune system to distinguish between pathogenic and commensal bacteria, thereby eliciting specific immune responses. Not all flagellins activate TLR5 equally. It is important to note that flagellin-TLR5 interactions are heterogeneous and exhibit a gradient of responsiveness. Thus, these proteins are classified based on their effect on TLR5-mediated signalling, which reflects their varied roles in immune evasion and host interactions [43,56].

#### Stimulator flagellins

Most studies on flagellin have primarily focused on its function as a virulence factor in pathogenic bacteria such as *S. enterica*, whose flagellins elicit robust binding and activation of TLR5. These flagellins are classified as “stimulator flagellins,” and their interactions with TLR5 are well-characterized [41]. Specifically, the D0 domain contains structural features that facilitate strong binding to preformed TLR5 dimers, thereby enhancing signalling capabilities [43]. TLR5 forms preassembled asymmetric homodimers even in the absence of flagellin, suggesting that ligand binding occurs at a pre-existing dimer interface and may involve the cooperative recognition of two flagellin molecules to establish an optimal response threshold [57]. This dimerization provides a structural basis for the highly sensitive and rapid activation of TLR5 signalling upon flagellin detection. The potent immune response triggered by these flagellins is crucial for host defence against infections. These proteins promote inflammation and the recruitment of immune cells, including neutrophils and macrophages. The allosteric binding site within the D0 domain facilitates this heightened response, as it significantly improves the efficiency of TLR5 signalling [43].

#### Silent flagellins

Certain commensal bacteria have evolved “silent flagellins” that bind TLR5 as effectively as stimulator flagellins but induce low TLR5 activation. This promotes immune homoeostasis and prevents excessive inflammation [43]. For instance, flagellins from commensal Lachnospiraceae species are less effective at activating TLR5. These proteins contribute to the maintenance of a balanced immune response and healthy gut microbiota [56,58]. They are recognized by TLR5 but fail to trigger a significant immune response. For instance, *Roseburia hominis* flagellin RhFlaB strongly binds to TLR5 but cannot activate it fully because it lacks an allosteric binding site in its D0 domain [43]. In contrast, *Salmonella*-derived FliC uses its D0 domain to form the 2:2 TLR5-flagellin functional complex required for full activation [43]. This “silent recognition” mechanism is crucial for maintaining immune tolerance of beneficial commensal bacteria. It enables the host to coexist with its microbiota without triggering unnecessary inflammatory responses that may disrupt gut homoeostasis. These flagellins are abundant in the human gut, particularly in non-industrialized populations [43]. Their modulation of immune responses emphasizes the role of flagellin in the co-evolution of host-microbe interactions. It further demonstrates the ability of this protein to balance immune evasion and adaptation to ensure persistent colonization with minimal disruption to the host.

#### Evader flagellins

Certain bacterial species have evolved “evader flagellins” that avoid both TLR5 binding and activation, thereby bypassing immune surveillance [43]. For instance, flagellins from the human pathogens *Helicobacter pylori* and *Campylobacter jejuni* evade TLR5 detection by altering the amino acid sequence in the N-terminal D1 domain while maintaining bacterial motility through compensatory mutations [59]. These modifications enable colonization of mucosal surfaces with reduced host immune activation, a strategy that likely contributes to their persistence and pathogenesis. The evolutionary significance of these flagellins lies in their ability to bypass immune surveillance mechanisms, thereby contributing to bacterial survival and host colonization.

The classification of flagellins based on TLR5 activity highlights the evolutionary race between bacterial evasion strategies and host immune detection mechanisms. Stimulator flagellins represent an ancient and conserved mechanism by which pathogens trigger a strong immune response, whereas silent and evader flagellins illustrate how bacterial flagellins have evolved mechanisms to ensure host tolerance. While this classification provides an important framework for understanding flagellin function, many mechanistic details of these interactions, including the presence and function of an allosteric binding site within the D0 domain, have been established in only a limited number of flagellins. This highlights that much remains to be investigated regarding the diversity of flagellin-TLR5 interactions across different bacterial species. This nuanced interaction between flagellins and TLR5 highlights the complexity of host-microbe interactions and the delicate balance that the immune system must achieve to protect against pathogens while tolerating beneficial microbes. These evolutionary adaptations provide insights into the mechanisms underlying microbial survival and the maintenance of host health.

This intricate co-evolution extends beyond immune modulation. It affects broader aspects of host physiology, including systemic inflammation and lipid metabolism. Dynamic interactions between flagellin and TLR5 underscore the role of the gut microbiota-TLR5 axis as a critical mediator of homoeostasis and disease pathogenesis. The comparative roles of TLR5 in the regulation of immune responses and metabolic processes across different host systems are summarized in Table 1. The following section explores this axis in detail, focusing on its role in host immunity.

**Table 1.** Summary of comparative immune and metabolic roles of TLR5 across host systems.

Host	System	Immune Role	Metabolic Role
Mice	Gut Immunity	TLR5 drives NF-κB activation and innate cytokine production (e.g. IL-6, TNF-α) to control microbial dysbiosis and prevent inflammation.	Regulates gut microbiota composition and maintains epithelial barrier integrity to maintain metabolic homoeostasis and prevent microbial translocation.
	Adaptive Immunity	Promotes flagellin-specific IgA and IgG production through MyD88-dependent pathways, enhancing mucosal and systemic protection.	IgA responses to flagellin influence the gut microbiota composition, modulation of SCFA production, and host metabolic regulation.
	Gut-Liver Axis	Facilitates IL-22 production by ILC3s and CD4+ T cells via IL-23 signalling to protect against gut-derived microbial translocation.	Hepatic TLR5 activation stimulates ApoA1 production, supporting HDL-C synthesis and lipid homoeostasis.
	Vaccine Efficacy	Enhances adaptive immunity to unadjuvanted vaccines (e.g. influenza) through TLR5-dependent plasma cell differentiation.	Flagellin-based vaccination protects against high-fat diet-induced obesity and inflammation by modulating the gut microbiota and reducing metabolic endotoxemia.
	Ageing	Maintains gut barrier integrity and promotes immune homoeostasis in aged mice via IL-22 and Reg3γ expression.	Delays age-associated metabolic dysregulation and systemic inflammation through gut microbiota modulation.
Humans	Genetic Variants	TLR5 polymorphisms (e.g. R392STOP) reduce flagellin-specific IgG/IgA responses and are negatively associated with Crohn’s disease.	The R392STOP variant is linked to reduced adiposity but increases susceptibility to type 2 diabetes.

Abbreviations: SCFA, short-chain fatty acid; ILC3s, RORγt+ type 3 innate lymphoid cells; HDL-C, high-density lipoprotein-cholesterol.

## The flagellin-TLR5 axis in host immunity

### TLR5: localization, structure, and immune activation

TLR5 is predominantly localized to the basolateral surface of gut epithelial cells. It is also expressed in immune cells, including dendritic cells (DCs) and macrophages [26,60,61]. This protein is a key component of the early detection of pathogenic invasion in innate immunity [62,63]. In reporter mouse models, TLR5 expression in the small intestine was predominantly restricted to Paneth cells within the intestinal crypts [64]. TLR5 is initially expressed broadly in the small intestinal epithelium during neonatal development. However, it gradually becomes restricted to Paneth cells as the gut matures [64]. This suggests that its developmental regulation is influenced by both intrinsic programming and early exposure to the flagellated gut microbiota. In contrast, TLR5 expression is more widespread in colonic epithelial cells; this reflects its diverse role in the intestinal tract [64]. Additionally, its broad distribution across the gut, liver, and lungs suggests its importance in the coordination of localized and systemic immune responses [65–67].

TLR5 detects bacterial flagellin, which activates innate immune responses through the MyD88-NF-κB pathway in monocytes and epithelial cells [60,68]. Structurally, TLR5 is composed of an extracellular leucine-rich repeat (LRR) ectodomain that recognizes bacterial flagellin, a transmembrane domain, and an intracellular toll/interleukin-1 receptor domain that triggers downstream signalling [69]. TLR5 undergoes dimerization upon binding flagellin to its LRR ectodomain, consequently initiating a signalling cascade that involves the adaptor protein MyD88 [41,56]. This MyD88-dependent pathway activates NF-κB, a transcription factor that induces the secretion of pro-inflammatory cytokines, such as IL-6, IL-12p40, TNF-α, and IL-1β. These cytokines facilitate the recruitment and activation of immune cells at the infection site, thereby promoting the clearance of pathogenic bacteria.

Dysregulation of TLR5 signalling can contribute to chronic inflammation in conditions such as IBD and metabolic syndrome. For example, the absence of TLR5-mediated signalling led to impaired regulation of systemic inflammation in TLR5-deficient mice [70,71]. Without TLR5, bacterial flagellin fails to activate the MyD88-NF-κB pathway, resulting in the reduced production of cytokines such as IL-6 and TNF-α. While this may lower inflammatory responses to the gut microbiota, it also leads to increased susceptibility to pathogenic infections and persistent low-grade inflammation; this contributes to systemic immune dysregulation. Moreover, the transfer of gut microbiota from TLR5-deficient mice to germ-free (GF) mice induced low-grade inflammation in recipients. This further indicates the importance of regulating the composition of flagellated gut microbiota [71].

### TLR5 in mucosal immunity and intestinal barrier integrity

In addition to its role in innate immune signalling, TLR5 regulates immune homoeostasis, particularly on mucosal surfaces. It recognizes bacterial flagellin and initiates pro-inflammatory pathways in mucosal DCs [72]. TLR5+ DCs promote Th1 and Th17 differentiation while suppressing regulatory T cell development, consequently maintaining an immune balance tailored to microbial threats [72]. Similarly, TLR5 signalling in DCs is essential for the production of IL-22, a cytokine that supports epithelial cell proliferation, antimicrobial peptide production, and tissue repair [73]. The loss of TLR5 in DCs impairs these processes, leading to an increased susceptibility to gut inflammation and systemic immune dysregulation [73]. Flagellins also activate TLR5 in intestinal epithelial cells, thereby inducing the secretion of IL-8 [50,60], a cytokine that recruits neutrophils to the infection site. This highlights the role of TLR5 in epithelial-driven early inflammatory responses [74]. Furthermore, these findings underscore the critical role of TLR5 in the modulation of immune homoeostasis through DC-mediated and epithelial-driven signalling.

Recent studies have further underscored the importance of TLR5 in the management of microbial composition and preservation of gut barrier integrity. For instance, TLR5/Rag1 double knockout (DKO) mice demonstrate severe immune dysregulation compared to their TLR5 knockout (T5-KO) and Rag1 knockout (Rag1-KO) littermates, with frequent lethal infections caused by Pasteurellaceae and increased abscess formation in multiple organs [75]. In TLR5/NLRC4/Rag1 triple knockout (TKO) mice, the loss of innate immune recognition mechanisms results in increased microbial encroachment into the inner mucus layer, disrupted gut barrier function, and dysbiosis characterized by elevated Proteobacteria levels, compared to Rag1-KO mice [75]. These mice also exhibited heightened susceptibility to *Citrobacter rodentium* infection, and severe colitis highlighted the interplay between innate and adaptive immunity in the management of gut homoeostasis [75]. These findings demonstrate that innate immune sensing of flagellin is critical not only for maintaining microbial balance but also for preventing inflammation and infections in immunocompromised states.

Within the mucosal environment, TLR5 signalling also promotes mucosal homoeostasis by inducing the expression of antimicrobial peptides, including RegIIIγ [76]. This C-type lectin is produced by intestinal epithelial and Paneth cells and exhibits bactericidal activity against gram-positive bacteria [77,78]. The activation of TLR5 by flagellin triggers a MyD88-dependent signalling cascade, leading to the production of IL-22 from RORγt+ innate lymphoid cells (ILCs) [79,80]. RegIIIγ expression is regulated by IL-22 and directly affected by the composition of the gut microbiota [81]. Systemic administration of flagellin restores innate immune defences compromised by antibiotic treatment, significantly enhancing RegIIIγ expression across the entire small intestine and limiting bacterial colonization [79]. These findings underscore the critical role of TLR5 in the regulation of antimicrobial defences and maintenance of microbial equilibrium in the gut.

Similar to its role in mucosal immunity, TLR5 activation also reinforces the gut barrier by preventing microbial translocation and mitigating systemic inflammation through interactions with commensal bacteria. This protective function is mediated by key mechanisms that preserve barrier homoeostasis, such as IL-22 signalling and antimicrobial peptide production. For instance, flagella from *Clostridia* induce IL-23 expression through TLR5 and MyD88 signalling in CD11c+ DCs. This stimulates IL-22 production from RORγt+ type 3 ILCs [82,83]. The IL-23-IL-22 axis strengthens the integrity of the intestinal barrier, reduces dietary allergen translocation, and promotes immune homoeostasis. A synergistic effect between TLR5 signalling and aryl hydrocarbon receptor (AhR) activation by Clostridial metabolites such as indole further enhances these protective mechanisms [83]. However, disruptions to commensal microbiota, such as those caused by neonatal exposure to antibiotics, can impair these pathways. This impairment leads to increased gut permeability and susceptibility to immune-mediated disorders, such as food allergies [82]. Increased allergic responses in mice deficient in TLR5 or AhR signalling further illustrate the therapeutic relevance of these pathways [83].

Flagellin from specific commensal bacteria, such as *Roseburia* spp., can further enhance the protective mechanisms of TLR5 and IL-22 in the gut barrier [84–86]. Mono-colonization of germ-free mice with *R. hominis* upregulated TLR5 expression, strengthened intestinal barrier integrity, and promoted immune tolerance mediated by flagellin-TLR5 signalling [84]. Similarly, flagellin from *Roseburia intestinalis* upregulates the expression of tight junction proteins such as Occludin and induces IL-22 expression through TLR5 activation [86]. Flagellin from *E. coli* Nissle 1917 activates TLR5, significantly increases IL-22 production, and prevents DSS-induced colitis [48]. These findings reinforce the concept of leveraging TLR5-mediated pathways to enhance the gut barrier function and control systemic inflammation.

### Adaptive immune responses to flagellin

Interaction between innate and adaptive immunity is critical for mounting an effective immune response against microbial antigens. Flagellin facilitates this interaction by activating TLR5-mediated pathways that are essential for T-cell priming and antibody production [87,88]. Unlike traditional thymus-independent antigens, flagellin requires activation of both innate immune cells and T cells to elicit specific antibody responses [25]. This process involves recognition by TLR5 or NLRC4 and further emphasizes the role of flagellin as a key bacterial antigen for immune regulation [30]. These receptors function redundantly to ensure that flagellin can stimulate antibody production even in the absence of one pathway [30]. The generation of flagellin-specific antibodies is MyD88-dependent, which indicates the essential role of innate immune activation in bridging adaptive immunity [25]. This dependency underscores the robustness of flagellin as an immunogenic molecule and its potential for adjuvant applications, further recognizing TLR5 as a key mediator in shaping humoral responses.

Systemic and mucosal antibodies constitute two distinct components of adaptive immunity. They are specifically adapted to protect different physiological compartments. Systemic antibody responses to gut microbiota-derived antigens play a critical role in host defences beyond the intestinal lumen. Pathogen-derived flagellins, such as FliC from *S. typhimurium*, are potent inducers of systemic antibody responses. This demonstrates the critical role of flagellin-specific antibodies in infection management and immune surveillance [89]. However, systemic anti-flagellin IgG response can exacerbate inflammation under certain conditions. In a murine model of colitis, anti-flagellin IgG antibodies enhanced inflammation following damage to the gut barrier [90]. This pathogenic effect is mediated by the neonatal Fc receptor (FcRn), which is expressed on myeloid cells. In addition, it enhances antigen presentation and adaptive immune activation [90]. FcRn-deficient mice were protected from colitis despite high circulating levels of anti-flagellin IgG, underscoring the pro-inflammatory role of this receptor [90].

IgG operates predominantly in the systemic compartments, whereas IgA serves as the principal antibody in mucosal environments and focuses on immune exclusion within the gut lumen. The predominant form of IgA on mucosal surfaces is secretory IgA (SIgA). In TLR5-deficient mice, the absence of TLR5 signalling led to significantly reduced levels of anti-flagellin IgA despite elevated total IgA levels in the gut [56]. This reduction in anti-flagellin IgA levels correlated with increased flagellin bioactivity and bacterial motility. This demonstrates the significance of TLR5-mediated immunity in the control of microbial dynamics and maintenance of mucosal barrier integrity [56].

Recent findings have highlighted the critical role of TLR5-expressing lamina propria DCs (LPDCs) in adaptive immune responses in the gut. These CD11c^hi^CD11b^hi^ LPDCs induce the differentiation of naïve B cells into IgA-secreting plasma cells independent of gut-associated lymphoid tissues and promote the generation of Th1 and Th17 cells in response to flagellin stimulation [91]. Retinoic acid produced by LPDCs further supports IgA production and gut-homing receptor expression. This indicates the importance of TLR5 in mucosal immunity [91].

These findings underscore the diverse roles of flagellin-specific immune responses in the maintenance of gut homoeostasis and systemic immune regulation. These interactions not only influence microbial dynamics and mucosal integrity but also have broader implications for metabolic health. The following section explores the role of the flagellin-TLR5 axis in linking immune signalling to metabolic homoeostasis, consequently highlighting its relevance in gut microbiota-driven metabolic regulation.

## The flagellin-TLR5 axis in host metabolism

### TLR5 and gut microbiota in metabolic homoeostasis

The ability of TLR5 to maintain the gut microbiota composition suggests that its dysfunction may exacerbate microbial dysbiosis, a common characteristic of metabolic disorders. For instance, TLR5-deficient mice exhibit increased fat deposition, systemic inflammation, and disrupted lipid metabolism, mirroring the features of metabolic syndrome in humans [71]. TLR5-deficient mice also exhibit transient instability in gut microbiota composition post-weaning, along with enrichment of Proteobacteria such as adherent-invasive *E. coli* (AIEC) [92]. This microbial dysbiosis correlates with colitis severity, indicating the protective role of TLR5 against gut inflammation and in the maintenance of microbial homoeostasis. Moreover, mice lacking TLR5 in intestinal epithelial cells (IECs) exhibit key features of low-grade inflammation, such as splenomegaly and elevated faecal levels of lipocalin-2, as well as hallmark characteristics of metabolic syndrome, including increased adiposity, hyperglycaemia, and dyslipidemia [73]. These phenotypes are strongly associated with altered microbial localization and increased levels of pro-inflammatory microbial products such as flagellin and lipopolysaccharide (LPS) in the gut. Furthermore, TLR5^ΔIEC^ mice showed disrupted gut microbiota composition characterized by an elevated Firmicutes/Bacteroidetes ratio, a microbial signature commonly linked to obesity [73,93].

Elevated TLR5 expression in adipose tissue is associated with obesity and metabolic dysfunctions in humans. Individuals with high TLR5 activity exhibit increased fat mass, dysregulated lipid metabolism, and elevated leptin levels [94]. These metabolic phenotypes are linked to gut dysbiosis, which is characterized by an increased abundance of flagellated bacteria, including *Clostridium* cluster XIV, and a higher Firmicutes-to-Bacteroides ratio [94]. Flagellin directly activated TLR5 signalling in adipocytes *in vitro*, leading to NF-κB activation, enhanced lipolysis, and impaired insulin signalling [94]. This highlights the dual role of TLR5 in the mediation of gut-adipose tissue crosstalk and underscores its potential as a target for addressing inflammation-driven metabolic disorders.

TLR5 expression in hepatocytes maintains gut-liver homoeostasis by facilitating bacterial clearance and mitigating microbial translocation-induced inflammation. The absence of hepatocyte TLR5 exacerbated diet-induced liver pathologies, including steatosis and fibrosis, by amplifying microbiota-driven inflammation in TLR5^ΔHep^ mice [66]. These findings suggest that targeting hepatocyte TLR5 or modulating gut microbiota may prevent metabolic liver diseases. Furthermore, a high-fat diet (HFD) increases the abundance of flagellated bacteria in the gut, and microbiota-derived flagellin activates hepatic TLR5 to stimulate the production of apolipoprotein A1, a critical component of high-density lipoprotein-cholesterol (HDL-C) [95]. In human cohorts, a positive correlation was observed between the flagellum-producing capacity of the gut microbiota and HDL-C levels, with sex-based differences observed [96]. This interaction involving species such as *R. intestinalis* and *Eubacterium rectale* highlights the therapeutic potential of targeting hepatic TLR5 to modulate lipid metabolism and mitigate atherosclerosis [96].

TLR5 signalling has also been implicated in liver regeneration. Research on partial hepatectomy (PHx) in mice revealed upregulated TLR5 expression in the liver during regeneration. This enhanced the hepatocyte proliferation and lipid accumulation necessary for repair processes [97]. Mice lacking TLR5 exhibit impaired liver regeneration due to reduced NF-κB and STAT3 activation and diminished production of pro-inflammatory cytokines that are crucial for initiating the regenerative process, such as TNF-α and IL-6 [97]. Conversely, the administration of the TLR5 agonist CBLB502 significantly enhanced liver regeneration by promoting cytokine production and immune cell recruitment [97]. These findings further highlight the multifaceted roles of TLR5 in immune regulation, metabolic homoeostasis, and tissue repair.

Genetic variations in human-derived TLR5 further illustrate its dual role in metabolic and immune regulation. For instance, the R392STOP single nucleotide polymorphism (SNP) terminates TLR5 function, thereby reducing its ability to recognize bacterial flagellin [98,99]. Carriers of the dominant-negative TLR5 polymorphism (TLR5-STOP) exhibit significantly reduced levels of flagellin-specific IgG and IgA, demonstrating a clear connection between innate recognition of flagellin via TLR5 and the regulation of adaptive immunity [100]. This polymorphism was associated with metabolic phenotypes in Jewish populations, including protection against obesity. Female carriers of this allele have a lower BMI and reduced adiposity, potentially due to diminished TLR5-mediated inflammatory signalling [101]. However, this protective effect against obesity is counterbalanced by the increased risk of type 2 diabetes mellitus, highlighting the complex relationship between TLR5 function and metabolic regulation [101]. This polymorphism is also negatively associated with Crohn’s disease in certain populations, highlighting its potential protective effect against chronic inflammatory disorders [100]. The metabolic effect of TLR5-flagellin interactions underscores the symbiotic relationship between the gut microbiota and the host. This provides insight into therapeutic strategies aimed at modulating TLR5 signalling to prevent or manage metabolic disorders.

### Mucosal anti-flagellin IgA and gut microbiota regulation

Mucosal anti-flagellin IgA modulates the microbial dynamics within the intestinal lumen. Although systemic antibodies contribute to host defences against flagellin and other microbial antigens that breach the gut barrier, mucosal IgA antibodies play a specialized role in the maintenance of gut homoeostasis. These antibodies exhibit a remarkable combination of cross-reactivity and selectivity [102]. Mucosal anti-flagellin IgA, primarily secreted in mucosal immune tissues such as Peyer’s patches and mesenteric lymph nodes, regulates the composition of the gut microbiota [89,103]. It is secreted into the intestinal lumen as part of the mucosal immune response and can be detected in faecal samples [56]. Anti-flagellin IgA specifically immobilizes flagellated bacteria and reduces their motility by downregulating the expression of flagellar motility genes [56]. These actions help maintain gut homoeostasis, prevent bacterial encroachment into the epithelial barrier, and reduce the pro-inflammatory potential of microbiota.

The levels of faecal anti-flagellin IgA are significantly reduced in obese individuals, correlating with higher bioactive faecal flagellin levels and increased bacterial motility [89]. This reduction in faecal anti-flagellin IgA is associated with disrupted gut microbiota and heightened intestinal inflammation, underscoring its critical role in the maintenance of microbial balance and prevention of metabolic dysregulation. The absence of TLR5 leads to reduced faecal anti-flagellin IgA levels, increased flagellin expression in gut microbiota, and breaches in the mucosal barrier [56]. Moreover, reduced mucosal IgA levels impair the ability of inducible costimulator ligand (ICOSL)-deficient mice to immobilize flagellated bacteria such as Lachnospiraceae, leading to dysbiosis and disrupted host-microbiota mutualism [104]. This imbalance underscores the dual role of Lachnospiraceae as beneficial commensals and potential pro-inflammatory agents when unregulated. The synergy between ICOSL-driven antibodies and IL-10 was evident in the mitigation of inflammation and maintenance of microbial balance, particularly during early life, when IgA responses complemented maternally derived antibodies to shape protective gut microbiota [104].

In addition to naturally occurring anti-flagellin IgA responses, active immunization strategies targeting flagellin have demonstrated considerable therapeutic potential. Systemic administration of purified *Salmonella* flagellin FliC induced robust anti-flagellin IgA and IgG responses in mice [89]. These responses reshape gut microbiota composition, prevent bacterial encroachment into the epithelium, and prevent colitis and HFD-induced obesity in mice. This demonstrates the therapeutic potential of targeting flagellin-specific immune pathways [89]. Moreover, flagellin immunization can prevent dietary emulsifier-induced dysbiosis and chronic inflammation by stabilizing IgA-microbiota interactions [105]. This immunization reduces the secretion of pro-inflammatory microbial products, including LPS and flagellin, and mitigates metabolic dysregulation, such as weight gain and hyperglycaemia [105]. These findings elucidate the potential of flagellin immunization as a protective strategy against microbiota-driven metabolic disorders. Moreover, these insights highlight the pivotal role of anti-flagellin IgA in orchestrating immune regulation of the gut microbiota, balancing microbial diversity, and maintaining mucosal homoeostasis.

### The effects of TLR5 and gut microbiota on healthspan

Ageing is associated with a marked reduction in TLRs function, leading to diminished secretion of cytokines, such as TNF-α, IL-6, and IL-12, and impaired activation of the innate immune pathways [106]. These changes weaken the critical link between innate and adaptive immunity. In addition, they contribute to an increased susceptibility to infection, chronic inflammation, and metabolic dysregulation. However, TLR5 expression and signalling are preserved in aged mice and older individuals. This presents a unique opportunity to mitigate immune decline and frailty through targeted therapies [107–109].

Recent studies have highlighted the profound impact of ageing on the composition of the gut microbiota and its implications for metabolic and immune health. Ageing is associated with a reduction in microbial diversity. This reduction is marked by a loss of beneficial taxa, such as Clostridiales and *Bifidobacterium*, and an increase in the abundance of pathogens such as Enterobacteriaceae species [110,111]. Dysbiosis, characterized by age-related loss of *Akkermansia muciniphila*, is associated with systemic inflammation and metabolic dysregulation, including impaired intestinal integrity, adiposity, and insulin resistance. This is mediated through the microbiome-monocyte-B cell axis [112].

Studies on the intestinal microbiota of elderly individuals have identified motile commensal bacteria, such as *E. rectale* and *Roseburia inulinivorans*, as prominent producers of flagellins with pro-inflammatory potential [58]. Flagellins from these species stimulate IL-8 secretion in intestinal epithelial cells, demonstrating their capacity to affect immune responses and contribute to localized inflammation. The abundance of these bacteria varies based on environmental and dietary factors, suggesting that microbial composition and metabolic activity in ageing populations are highly dynamic. These findings highlight the dual role of commensal-derived flagellins in supporting gut homoeostasis and modulating immune responses in elderly populations.

The role of the microbiota in age-related immune decline extends to the regulation of M cell density in Peyer’s patches. The transfer of microbiota from young mice restored M cell maturation and improved antigen uptake and mucosal IgA responses in aged mice, further highlighting the critical role of healthy microbiota in intestinal immunity [113]. Additionally, systemic administration of flagellin increased M cell density and enhanced immune responses. This provides a promising therapeutic avenue for the mitigation of age-associated decline in intestinal immunity [113]. These interventions have been linked to specific microbial composition changes, including increased *A. muciniphila* and reduced *Turicibacter* spp. abundance, further underscoring the interplay between microbiota composition and mucosal immune homoeostasis [113].

Recent studies have highlighted the potential of mucosal TLR5 activation as a therapeutic strategy against age-associated immune defects. For instance, nasal delivery of a flagellin-containing fusion protein extended the healthspan and lifespan of aged mice. It reduced age-related conditions such as hair loss, cataracts, and pulmonary fibrosis [114]. This intervention enhances intestinal mucosal integrity by increasing TLR5 expression in LPDCs and promoting IL-22 secretion, which is critical for epithelial regeneration and immune regulation [114]. Furthermore, faecal SIgA levels were elevated, thereby contributing to enhanced gut barrier function and microbial balance [114]. Despite increased food intake, treated aged mice exhibited stable body weight and metabolic homoeostasis, along with reduced markers of systemic inflammation and senescence in major organs such as the liver and intestines [114]. These findings emphasize the dual role of TLR5 in intestinal homoeostasis and metabolic regulation. TLR5 activation prevents age-related decline in mucosal immunity and promotes tissue regeneration. Therefore, it is a promising therapeutic avenue for addressing age-associated metabolic disorders and inflammation-driven diseases.

## Therapeutic and diagnostic applications of flagellin

The adjuvant ability of flagellin to activate TLR5 makes it an excellent candidate for enhancing vaccine efficacy. As a natural ligand of TLR5, flagellin stimulates innate immune responses that bridge adaptive immunity and enhance the production of antigen-specific antibodies with minimal safety concerns [24]. Targeting TLR5 using agonists has shown promise in the mitigation of chronic inflammation and metabolic disturbances. Moreover, the therapeutic potential of TLR5 activation extends beyond vaccination. Recent studies have explored the application of TLR5 agonists and engineered delivery systems under various conditions.

### Enhancement of influenza vaccine efficacy

The unique properties of flagellin make it a promising tool for vaccine development, as demonstrated by flagellin-based hybrid vaccines that provide robust protection against influenza. Flagellin offers advantages over traditional adjuvants, such as LPS, owing to its low toxicity and unique ability to activate epithelial cells [24]. This activation enhances the upregulation of the expression of cytokines and cytoprotective genes, thereby providing non-specific protection against respiratory pathogens [115]. The plasticity of this protein also allows it to act as a fusion protein that physically links antigens to its structure, or as a co-administered adjuvant to augment immune responses [116,117].

Although some studies suggest that antigen linking is necessary for optimal adjuvant activity, others have shown that co-administration can also produce potent immunity [87,116]. For instance, a fusion of flagellin with haemagglutinin (HA) from the H1N1 influenza virus (VAX125) demonstrated enhanced immunogenicity in preclinical and clinical studies, providing superior protection against influenza infections [107,118]. Modified designs, such as those replacing the hypervariable region of flagellin with the HA globular head of H5N1, have proven even more effective, eliciting stronger and longer-lasting neutralizing antibody responses than conventional vaccine formulations [119].

Recent studies have explored the potential application of flagellin in the development of COVID-19 vaccines. A notable example is a DNA vaccine designed to encode the SARS-CoV-2 S1 protein fused with optimized flagellin. This vaccine significantly enhanced immunogenicity in murine models [120]. The formulation induced robust and balanced Th1/Th2 immune responses and long-lasting humoral immunity, consequently outperforming non-flagellin-based formulations [120]. The applicability of flagellin as a vaccine adjuvant for both influenza and SARS-CoV-2 indicates its versality. Moreover, flagellin demonstrated effective adjuvant activity across diverse age groups, including the elderly, who often respond poorly to traditional vaccines. Flagellin-based adjuvants enhanced both systemic and mucosal antigen-specific immune responses in aged mice, albeit at slightly reduced levels compared to those in younger mice [121,122].

The broad applicability of flagellin as an adjuvant extends beyond its direct immunogenic properties to its ability to modulate the gut microbiota, which is a critical factor in vaccine-induced immunity. TLR5-mediated sensing of the gut microbiota is essential for effective antibody responses, as flagellin recognition through TLR5 promotes plasma cell differentiation and enhances humoral immunity to unadjuvanted vaccines, including the trivalent inactivated influenza vaccine (TIV) [123]. Mice lacking TLR5 or treated with antibiotics exhibit reduced antibody responses to TIV. This indicates the importance of a healthy gut microbiota in vaccine efficacy [123]. These findings highlight the potential use of gut microbiota-derived flagellin to boost vaccine-induced immunity. Furthermore, these findings suggest that flagellin-based adjuvants are uniquely positioned to enhance influenza vaccine efficacy by boosting both systemic and mucosal immunity. In addition, these adjuvants provide a safer and more versatile alternative to conventional protein-based adjuvants. This underscores their potential as critical tools for improving vaccine response against influenza and related pathogens.

### Cancer immunotherapy

TLR5 has emerged as a promising target in cancer immunotherapy, owing to its ability to activate both innate and adaptive immune responses. For instance, an engineered *S. typhimurium* strain that secretes *Vibrio vulnificus* flagellin B (VvFlaB) directly into tumours has been developed [124]. This system targets hypoxic tumour regions, where bacteria proliferate and release VvFlaB, thereby activating TLR5 in the immune cells. TLR5 activation by VvFlaB initiates a cascade of immune responses, including the recruitment of neutrophils and macrophages, polarization of macrophages from the immunosuppressive M2 phenotype to the pro-inflammatory M1 phenotype, and enhanced secretion of cytokines such as TNF-α and IL-1β [124]. This shift creates a pro-inflammatory tumour immune microenvironment that suppresses tumour growth and inhibits metastasis [124]. The therapeutic effects of this system were diminished in TLR5-deficient mice, indicating the critical role of TLR5 in the mediation of immune responses [124].

VvFlaB was further combined with dual-drug payloads delivered via *S. typhimurium* [125]. VvFlaB-mediated TLR5 activation polarizes macrophages from the immunosuppressive M2 phenotype to the pro-inflammatory M1 phenotype, consequently enhancing CD8+ T cell infiltration and anti-tumour cytokine secretion [125]. These effects transformed “cold” tumours into “hot” tumours characterized by robust immune activity and sustained tumour regression [125]. This strategy also showed synergistic potential when combined with immune checkpoint inhibitors; this combination amplified the overall anti-tumour response.

The genetic variability of TLR5 in humans affects cancer progression and immune surveillance. Specific missense SNPs in TLR5, such as rs5744174 (F616L) and rs2072493 (N592S), affect human colorectal cancer survival by modulating the immune response to bacterial flagellin [126,127]. For instance, the F616L variant reduces TLR5 responsiveness. This leads to reduced levels of pro-inflammatory cytokines and consequently prevents colorectal cancer progression [126]. These findings further highlight the importance of TLR5 in shaping tumour immune dynamics and its potential as a therapeutic target in oncology.

### The diagnostic potential of systemic anti-flagellin antibodies

Advancements in high-throughput antibody profiling technologies, such as Phage Immunoprecipitation Sequencing, enable the comprehensive mapping of systemic antibody responses to gut microbiota-derived antigens [128]. Systemic antibody repertoires to gut microbiota-derived antigens are remarkably more stable over time than the gut microbiota species composition. In addition, they served as robust immunological fingerprints in a cohort of 997 healthy individuals [128]. Flagellin has emerged as a crucial target for both IgG and IgA antibody responses, reflecting its immunogenicity and importance in host-microbe interactions.

Altered systemic antibody responses to commensal flagellin have been observed in chronic inflammatory diseases, which indicates their diagnostic potential [32,100,129]. Crohn’s disease (CD), characterized by a dysregulated immune response to the gut microbiota, provides a unique context in which specific flagellins can serve as predictive biomarkers [129]. CBir1, a flagellin identified as a dominant antigen in patients with CD, is phylogenetically related to flagellins from *Clostridium* cluster XIVa [129,130]. Although CBir1 was originally identified in the caecal microbiota of colitic mice, its relevance in human CD is supported by the presence of CBir1-specific IgG antibodies in patients with CD [129]. These antibodies were not observed in patients with UC or in healthy controls, suggesting that CBir1 or structurally similar flagellins act as immunodominant antigens in CD.

Flagellins derived from Lachnospiraceae elicit strong serum IgG responses, particularly in CD [32]. Contrary to those with UC or healthy controls, patients with CD exhibited elevated reactivity to flagellins from human-derived *Roseburia* and *Eubacterium* species [32]. These responses were linked to heightened adaptive immunity, with multi-flagellin reactivity correlating with elevated levels of flagellin-specific CD4 T effector memory cells and a reduced regulatory-to-effector T cell ratio [32].

Systemic antibody responses to Lachnospiraceae flagellins have also been observed in other conditions involving immune dysregulation, such as myalgic encephalomyelitis/chronic fatigue syndrome (ME/CFS), in which elevated antibody reactivity distinguishes patients from healthy individuals [131]. The combination of antibody response profiles with traditional blood test data using machine learning models improved the diagnostic accuracy of ME/CFS [131]. In addition to inflammatory diseases, patients with short bowel syndrome (SBS) exhibit elevated systemic antibody responses to flagellin, owing to gut barrier dysfunction [132]. Patients with SBS have significantly increased levels of serum anti-flagellin IgM, IgA, and IgG. This indicates systemic exposure to bacterial products, such as flagellin and LPS [132]. These findings demonstrate that gut barrier failure can amplify systemic immune responses, further underscoring the diagnostic potential of anti-flagellin antibodies as markers of barrier integrity and immune activation.

These studies highlight the diagnostic potential of systemic anti-flagellin antibodies as biomarkers and therapeutic targets for chronic microbiota-related diseases in humans. Moreover, these findings underscore the importance of systemic antibody responses in reflecting immune activation, gut barrier dysfunction, and disease states. Thus, they provide valuable insights into diagnostic and therapeutic development.

## Concluding remarks and future perspectives

The intricate interplay between bacterial flagellin and host immunity, particularly through TLR5 signalling, highlights the evolutionary balance between microbial survival strategies and host defence mechanisms. This review highlights the pivotal roles of flagellin in immune modulation, ranging from its structural interaction with TLR5 to its therapeutic applications. Our literature survey indicates that this bacterial protein has significant implications for human health. Moreover, the discovery of “silent” and “evader” flagellins provides insights into the mechanisms underlying the adaptation of commensal and pathogenic bacteria to the host environment, potentially shaping the development of future microbiome-based therapies. Bacterial genomes often harbour multiple flagellin genes, each encoding a protein with varying TLR5 activation potential [43,84]. This diversity suggests that bacteria can selectively express specific flagellins in response to environmental stimuli such as host-derived signals or dietary components. Investigating these adaptive mechanisms can provide valuable insights into how bacteria balance their immune stimulation and evasion in dynamic host environments. The dual function of flagellin, as both a potent immune activator and a tool for microbial evasion, offers critical insights into host-microbe co-evolution. Thus, future research should focus on the following aspects:

### Expanding insights into flagellin variability

The structural diversity of flagellin, particularly in its hypervariable regions, reflects an evolutionary race driven by the need to maintain motility while evading immune detection. Thus, future research should prioritize comprehensive structural studies to elucidate the molecular mechanisms underlying flagellin-mediated immunomodulation in diverse bacterial species.

### Addressing host-specific responses

Host-specific variations in TLR5 expression and recognition add another layer of complexity to the flagellin-host interactions. For instance, the diversity of TLR5 ectodomain structures across species leads to differential binding affinities for bacterial flagellins, which can substantially affect immune signalling pathways [133]. Investigating these differences, particularly the distinct characteristics of TLR5 between mice and humans, will provide critical insights into how host genetics and microbiota composition shape immune responses, consequently paving the way for the development of personalized therapeutic strategies.

### Exploring environmental modulators of flagellin expression

Environmental factors such as dietary components or host mucus glycans serve as growth substrates that can significantly influence the expression of bacterial genes, including flagellin [134]. For instance, bacterial genomes often encode multiple flagellins with distinct TLR5 stimulatory abilities. Understanding how these genes are selectively expressed in response to specific environmental cues could reveal novel microbiome-host interaction dynamics.

### Harnessing microbiome diversity for therapeutics

Leveraging the diversity of gut microbiota-derived flagellin is a promising avenue for the development of next-generation probiotics. By identifying “silent” or “evader” flagellins, future interventions could fine-tune immune responses to promote health and resilience against diseases.

### Flagellin as an immunotherapeutic tool

Engineered flagellins have immense potential for therapeutic applications, including immunotherapy for chronic inflammatory conditions and ageing. Their capacity to selectively modulate TLR5 signalling offers a strategic approach for restoring immune balance while mitigating overactive inflammatory pathways.

In conclusion, the integration of structural biology, immunology, and microbiome research is required to explore the therapeutic potential of flagellin. This multidisciplinary approach can facilitate the comprehensive characterization of host-microbe interactions and development of innovative strategies for managing health and disease.

## Data Availability

Data sharing is not applicable to this article as no data were created or analysed in this study.
